# AAV-Mediated Delivery of Zinc Finger Nucleases Targeting Hepatitis B Virus Inhibits Active Replication

**DOI:** 10.1371/journal.pone.0097579

**Published:** 2014-05-14

**Authors:** Nicholas D. Weber, Daniel Stone, Ruth Hall Sedlak, Harshana S. De Silva Feelixge, Pavitra Roychoudhury, Joshua T. Schiffer, Martine Aubert, Keith R. Jerome

**Affiliations:** 1 Vaccine and Infectious Disease Division, Fred Hutchinson Cancer Research Center, Seattle, Washington, United States of America; 2 Department of Laboratory Medicine, University of Washington, Seattle, Washington, United States of America; 3 Division of Allergy and Infectious Disease, Department of Medicine, University of Washington, Seattle, Washington, United States of America; 4 Clinical Research Division, Fred Hutchinson Cancer Research Center, Seattle, Washington, United States of America; 5 Department of Microbiology, University of Washington, Seattle, Washington, United States of America; Drexel University College of Medicine, United States of America

## Abstract

Despite an existing effective vaccine, hepatitis B virus (HBV) remains a major public health concern. There are effective suppressive therapies for HBV, but they remain expensive and inaccessible to many, and not all patients respond well. Furthermore, HBV can persist as genomic covalently closed circular DNA (cccDNA) that remains in hepatocytes even during otherwise effective therapy and facilitates rebound in patients after treatment has stopped. Therefore, the need for an effective treatment that targets active and persistent HBV infections remains. As a novel approach to treat HBV, we have targeted the HBV genome for disruption to prevent viral reactivation and replication. We generated 3 zinc finger nucleases (ZFNs) that target sequences within the HBV polymerase, core and X genes. Upon the formation of ZFN-induced DNA double strand breaks (DSB), imprecise repair by non-homologous end joining leads to mutations that inactivate HBV genes. We delivered HBV-specific ZFNs using self-complementary adeno-associated virus (scAAV) vectors and tested their anti-HBV activity in HepAD38 cells. HBV-ZFNs efficiently disrupted HBV target sites by inducing site-specific mutations. Cytotoxicity was seen with one of the ZFNs. scAAV-mediated delivery of a ZFN targeting HBV polymerase resulted in complete inhibition of HBV DNA replication and production of infectious HBV virions in HepAD38 cells. This effect was sustained for at least 2 weeks following only a single treatment. Furthermore, high specificity was observed for all ZFNs, as negligible off-target cleavage was seen via high-throughput sequencing of 7 closely matched potential off-target sites. These results show that HBV-targeted ZFNs can efficiently inhibit active HBV replication and suppress the cellular template for HBV persistence, making them promising candidates for eradication therapy.

## Introduction

Hepatitis B virus (HBV) remains a significant cause of morbidity and mortality worldwide [1]. Long-term chronic infection with HBV carries a poor prognosis as it frequently leads to the development of cirrhosis and hepatocellular carcinoma (HCC) [2]. For those unable to spontaneously clear HBV infection, antiviral drugs can be used to inhibit viral replication, delaying long-term liver damage [3]. Unfortunately, HBV reactivation is common due to the persistence in hepatocytes of episomal covalently closed circular DNA (cccDNA), the template for HBV viral replication and the source for viral reactivation. In chronically infected individuals, cccDNA is able to persist, and while antiviral therapies can reduce hepatic cccDNA they do not entirely eliminate it [4].

Curative therapy strategies for chronically infected patients should disrupt or eliminate residual hepatic cccDNA, and thus prevent HBV reactivation. One potential approach involves eliminating or modifying viral sequences enough to sufficiently disrupt HBV gene functions. This could be achieved by introducing double strand breaks (DSBs) into viral DNA using targeted endonucleases. DNA DSBs are repaired through the process of non-homologous end joining, which is error prone, and repeated DNA cleavage will eventually yield mutations at a targeted cleavage site [5]. Alternatively, episomal DNA that is linearized from DSBs may be susceptible to direct degradation by cellular DNases, which could result in reduced viral replication or virus elimination. This strategy of targeted gene disruption has been proposed as a novel anti-viral therapy [6]–[8]. Indeed, several DSB-inducing enzymes, including zinc finger nucleases (ZFNs), transcription activator-like effector nucleases (TALENs), homing endonucleases (HEs), and the CRISPR/Cas system could be used in such an approach.

High specificity is a requirement of targeted endonucleases so as to avoid toxicity and other negative outcomes caused by the cleavage of genomic sequences with similarity to the target sequences, known as “off-target” sites. Off-target activity of endonucleases depends on their cleavage efficiency, the length of the target sequence, and the ability to recognize the correct sequence with high specificity [9]. Efforts are being made to improve these characteristics for some of the targeted endonuclease platforms [10]. Several in-depth analyses focused solely on off-target activity of targeted endonucleases have been performed [11], particularly for the CRISPR/Cas system [12]–[14]. Indeed, it is essential to assay the off-target activity of enzymes that possess successful mutagenic capability at their intended sites, especially in the case of antiviral enzymes that might be systemically delivered and thus expressed in a large number of cells. There exist bioinformatics programs for identifying potential off-target sites [12], [15], and high-throughput sequencing methods allow for many sites to be queried for potential mutagenesis following treatment with endonucleases.

Several reports have shown that targeted endonucleases can disrupt viral DNA sequences from HBV, HIV, HPV, HSV and HTLV [16]–[21]. In fact, antiviral effects against HBV have been achieved through the use of ZFNs [17] and TALENs [22], [23]. The next steps in preparing a robust antiviral therapy based on virus-specific targeted endonucleases for clinical application involve the development of a practical and efficient delivery method and a clear absence of off-target activity.

## Materials and Methods

### Cell culture

Human embryonic kidney (HEK) 293T cells and HepAD38 cells [24] were grown in DMEM (Invitrogen) supplemented with 10% FBS. HepAD38 cells are derived from HepG2 and can replicate HBV from a single integrated 1.1 length copy of a genotype D *ayw* serotype HBV genome under the control of the tetracycline responsive (tet-off) promoter. HBV replication in HepAD38 cells was suppressed in some experiments by the addition of 0.5 µg/ml doxycycline (dox). HepAD38 cells were grown on poly-L-lysine coated plates.

### ZFN target site heterogeneity analysis

A list of 3847 Genbank HBV genome sequences from the Hepatitis Virus Database (http://s2as02.genes.nig.ac.jp/) was aligned using clustalx [25]. A phylogenetic analysis of the HBV clustal alignment was performed with GeneiousPro (www.geneious.com) and 440 genotype A, 2233 genotype B or C, and 674 genotype D sequences were identified based on sequence clustering. A clustalx alignment of either the 3847 total sequences or the separate genotype (A, B&C, or D) sequences was used for sequence heterogeneity studies of the 3 ZFN target sites, and logo plots were generated using GeneiousPro.

### Zinc finger nucleases

**Figure 1:**
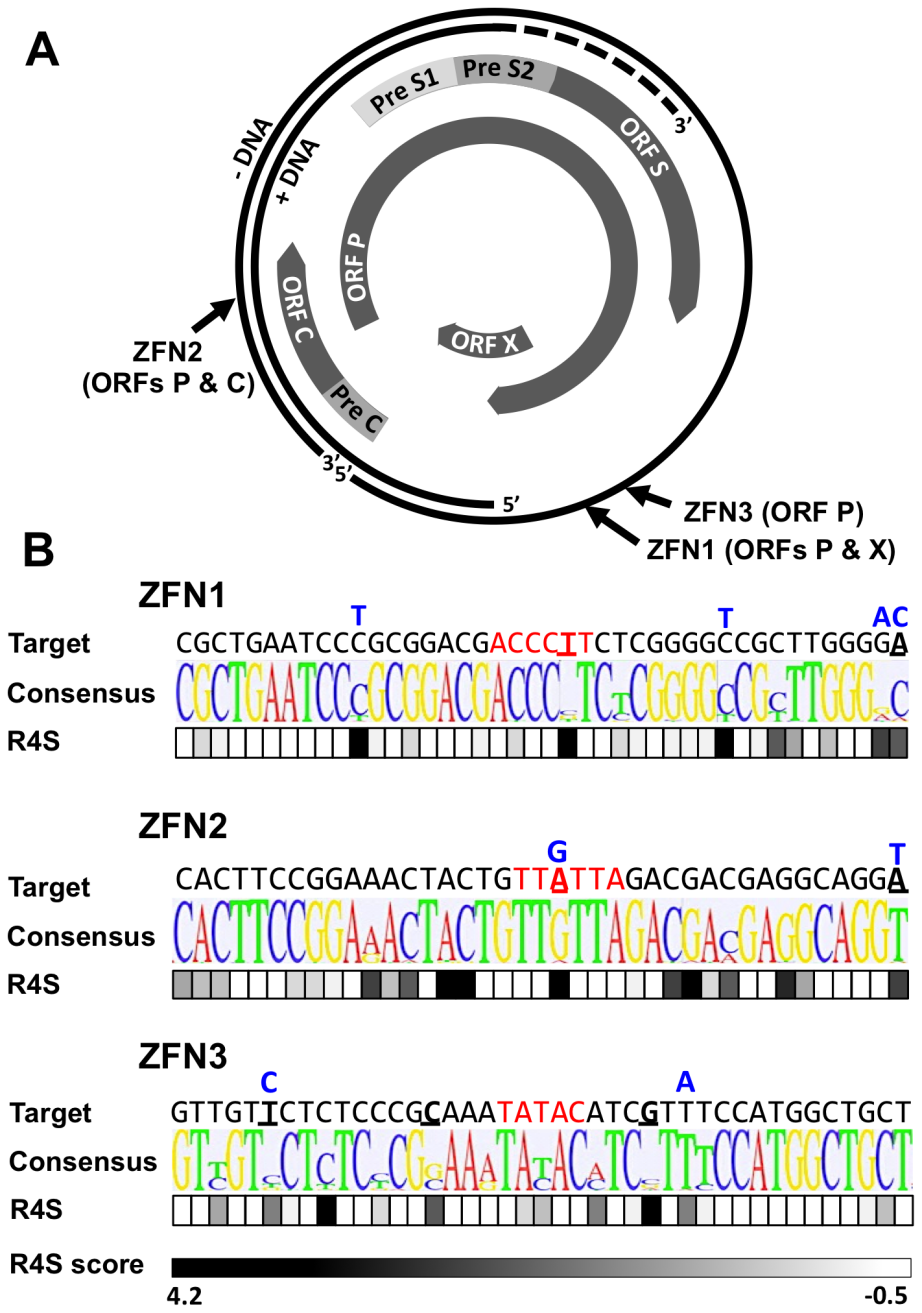
HBV-ZFN target sites. (***a***) HBV rcDNA genome map showing HBV ORFs and ZFN target site locations. (***b***) HBV target site sequence heterogeneity for HBV-ZFN pairs 1–3 across 3847 complete HBV genotype A–H sequences found in Genbank. For each ZFN pair, the target sequence, consensus sequence logo plot and nucleotide Rate4Site (R4S) scores are shown. ZFN spacer nucleotides are highlighted in red and divergent nucleotides between the ZFN target site and the consensus sequence are bold and underlined. Rate4Site scores are graded from low (white) to high (black) sequence heterogeneity. Single nucleotide polymorphisms present in the HepAD38 genomic HBV sequence are shown above each target site in blue. ORF – open reading frame; R4S – Rate4Site; ZFN – zinc finger nuclease.

Zinc finger nuclease pairs that target HBV sequences in open reading frame (ORF) P/ORF X (ZFN1), ORF P/ORF C (ZFN2) and ORF P (ZFN3) were custom generated by Sigma Life Science. To minimize off-target effects, each ZFN pair requires FokI heterodimerization for DNA cleavage [26]. Genomic locations and target sequences for ZFNs 1–3 are shown (Figure 1A, B). Each ZFN showed target site cleavage activity in a yeast-based assay (data not shown).

### *In vitro* ZFN activity assay

Target sites for ZFN1, ZFN2 and ZFN3 were cloned into the EcoRI/BamHI sites of the plasmid pRRL.SFFV.RS-GFP as previously described [16]. Target sites were placed between the green fluorescent protein (GFP) Turbo start codon and the GFP ORF to knock down GFP expression through gene disruptions that result in frame-shift mutations. The PEST amino acid sequence from mouse ornithine decarboxylase was fused to the C-terminus of the GFP gene in order to cause rapid proteasomal degradation of the GFP resulting in enhanced protein turnover, and sensitive detection of changes in gene expression [27]. Following treatment with a proteasome inhibitor, MG132 (Calbiochem), non-mutated, fully expressed GFP can be visualized in cells due to accumulation. A total of 4.5×10^5^ HEK 293T cells were plated in 12-well plates. The following day cells were transfected with 500 ng of each reporter plasmid with or without 1000 ng of each ZFN expression plasmid pair (pCMV.ZFNA.BGHpA & pCMV.ZFNB.BGHpA) using polyethylenimine (PEI) (1 mg/ml) (PolySciences, Inc.) at a 4∶1 (µl PEI:µg plasmid DNA) ratio. At 72 hours post-transfection, 1 µM MG132 was added and 4 hours later GFP expression was analyzed by microscopy.

### Polymerase chain reaction (PCR) amplicon sequence analysis

For analysis of gene disruption in HEK 293T or HepAD38 cells, DNA was isolated using the DNeasy Blood & Tissue Kit (Qiagen). ZFN target sites were amplified using Platinum *Pfx* DNA polymerase (Invitrogen) and PCR primers flanking the target sequence (Table S1). PCR products were then sub-cloned using the Zero Blunt TOPO PCR cloning kit (Invitrogen). HBV-ZFN target sites present in the plasmids pRRL.SFFV.ZFN1-GFP, pRRL.SFFV.ZFN2-GFP and pRRL.SFFV.ZFN3-GFP were amplified using primers SFFV-F and TurboGFP-R (Table S1). HBV-ZFN target sites present in the integrated HBV sequence within HepAD38 cells were amplified using PCR primers ZFN1/3-F and ZFN1/3-R for target sites ZFN1 and ZFN3, or PCR primers ZFN2-F and ZFN2-R for target site ZFN2 (Table S1). TOPO-cloned PCR products were transformed into One Shot Top10 *Escherichia coli* (Invitrogen) for clonal analysis and individual colonies were picked for plasmid purification from which the clonal ZFN target sites were sequenced using T7 or SP6 sequencing primers.

### DNA mutagenesis detection

DNA isolated using the DNeasy Blood & Tissue Kit (Qiagen) was used to amplify PCR products for ZFN sites 1 & 3, or ZFN site 2 using Platinum *Pfx* DNA polymerase and primers listed in Table S1. The Surveyor nuclease assay was performed using the Surveyor Mutation Detection Kit (Transgenomic, Omaha, NE) according to the manufacturer's instructions. NcoI digestion was used to detect the disruption of an NcoI site (CCATGG) contained in ZFN target site 3. This was done by adding 1 µl NcoI (New England Biolabs) and 1 µl NEB buffer #4 to 8 µl PCR amplicon, and incubating at 37C for 1 hr. Gel fragments were visualized on 2% agarose gels and band intensities were quantified using ImageJ software.

To evaluate cccDNA, DNA was isolated using a modified HIRT procedure [28] and digested with ATP-dependent Plasmid-safe DNase (Epicenter Biotechnologies, Madison, WI) according to the manufacturer's instructions.

### scAAV vector plasmids

The plasmid pscAAV-CMV-GFP (previously called pscAAV-GFP) expresses humanized Renilla reniformas GFP (hrGFP) from the human cytomegalovirus (hCMV) promoter and has been described before [29]. The plasmid pscAAV-EFS-GFP was generated by PCR amplifying a previously described short EF1α (EFS) promoter [30] using the primers EFS-F and EFS-R (Table S1) and cloning it as an AscI-XhoI fragment into the plasmid pscAAV-eGFP [31] to replace the full length EF1α promoter. The plasmid pscAAV-EFS-pA was generated by deleting the Acc65I-BsrGI eGFP fragment from the plasmid pscAAV-EFS-GFP. The plasmid pscAAV-EFS-mCherry was generated by PCR amplifying the mCherry gene with primers mCherry-1 and mCherry-2 (Table S1) and cloning it as a XhoI-NotI fragment into pscAAV-EFS-pA. HBV-ZFN-expressing scAAV vectors pscAAV-EFS-ZFN1A, pscAAV-EFS-ZFN1B, pscAAV-EFS-ZFN2A, pscAAV-EFS-ZFN2B, pscAAV-EFS-ZFN3A and pscAAV-EFS-ZFN3B were generated by cloning HBV-ZFN PCR fragments into the plasmid pscAAV-EFS-GFP as HindIII/NotI fragments. PCR fragments for HBV-ZFN1A, 2A & 3A were amplified using primers ZFN-F and ZFN-R1 (Table S1). PCR fragments for HBV-ZFN1B, 2B & 3B were amplified using primers ZFN-F and ZFN-R2 (Table S1).

### Production of AAV vectors

scAAV vectors were generated by transiently transfecting HEK 293T cells using PEI according to the method of Choi et al [32]. Briefly, HEK 293T cells were transfected with a scAAV vector expression plasmid, a plasmid that expresses the AAV capsid protein (pRepCap) and a helper plasmid that expresses adenovirus helper proteins (pHelper). pRepCap plasmids that contain the AAV cap gene from serotypes 1, 2, 4, 6, 8 or 9 were used to package scAAV vectors. At 72 hours post-transfection, cells were collected and re-suspended in AAV lysis buffer (50 mM Tris, 150 mM NaCl, pH 8.5) before freeze-thawing 4 times. AAV lysates were filtered through a 0.45 µm filter and stored at −80°C.

### scAAV quantification

All scAAV vector stocks were quantified by quantitative PCR (qPCR) according to the method of Fagone et al [33] using linearized plasmid DNA as a standard. scAAV reporter viruses were titered using primers that amplify regions of hrGFP, eGFP or mCherry genes (Table S1). scAAV HBV-ZFN viruses were titered using primers that amplify a conserved region of the FokI domain within each HBV-ZFN (Table S1).

### scAAV transduction analysis

A total of 5×10^5^ HepAD38 cells were seeded in 6-well plates. The following day, indicated scAAV vectors were added to cells at a multiplicity of infection (MOI) ranging between 100 and 5000 vector genomes/cell. Transduction of infected cells was analyzed by flow cytometry and fluorescence microscopy for GFP and/or mCherry expression at 72 hours post-infection.

### ZFN toxicity and viability assays

A total of 4×10^4^ HepAD38 cells were seeded in 96-well plates. Two days later they were treated with indicated ZFN-expressing or control scAAV vectors at a total MOI of 10000 vector genomes/cell. In samples with all three ZFN pairs combined, the total scAAV MOI was 10000 (low) or 30000 (high) vector genomes/cell. In samples treated with one ZFN subunit alone the total MOI was 5000 genomes/cell. At 48 hours post-transduction, cell viability was measured by MTT assay (Cayman Chemical) according to the manufacturer's instructions. Controls were untreated cells and cells treated with 0.1% Triton X-100. For a time-course assay on the effect of scAAV-ZFN treatment on cell viability, 1×10^6^ HepAD38 cells were plated in 60 mm dishes. The following day, cells were treated with indicated ZFN-expressing or control scAAV vectors at a total MOI of 10000 vector genomes/cell and dox was removed. At days 1, 3, 5, 7, 9, 11, and 14, cells were photographed and passaged to new dishes with equivalent numbers of cells plated for each treatment group, except for samples in which the treatment was significantly toxic (Z2A/Z2B, Z2A/Z3B), in which case the entire cell fraction was passaged to a new dish.

### Off-target analysis

The off-target predictor program PROGNOS [15] was utilized to determine the closest potential off-target sites in the human genome. The top two off-target sites for ZFN1A/B, top three sites for ZFN2A/B, and top two sites for ZFN3A/B were chosen for off-target analysis (Table S2). PCR primers (Table S1) were designed to amplify each of the seven off-target sites from HepAD38 cells that had been treated with the corresponding scAAV-ZFN pairs, as well as the on-target HBV sites. PCR amplicons were purified with a PCR clean-up kit (QIAgen), pooled together in equimolar ratios, and sequenced by single molecule real time sequencing (SMRT) (Pacific Biosciences) at the University of Washington PacBio Sequencing Services (https://pacbio.gs.washington.edu/). 15,437 reads filtered for size and FASTQ quality score (SMRT Portal software, Pacific Biosciences) were aligned to the 10 reference sequences (GeniousPro) in order to detect targeted mutagenesis in the ZFN target sites and potential off-target sites. An indel was defined as a deletion and/or insertion of at least 2 nucleotides (nt) in the spacer region of the target site. The average FASTA quality score per nt (in ASCII encoding corresponding to Phred scores) [34] was calculated for each read. Reads with an average score less than 80 (max ASCII value 113 over all reads) were deemed low quality (LQ) and removed. Reads that aligned poorly with the reference sequence (too short, too long, or possessing differences from the reference homogenously distributed across the entire read) were removed. Finally, of the remaining reads, any that possessed a single nt insertion, deletion or substitution located in the spacer region were excluded. This was because single nt indels could not be distinguished as homopolymer sequencing artifacts or byproducts of ZFN activity or PCR amplification. The sequencing data has been archived in the NIH Short Read Archive (accession SRP040654).

### HBV replication studies

HepAD38 cells grown in the presence of dox for 14 days were treated with HBV-ZFN-expressing scAAV2 vectors (total MOI 10000 vector genomes/cell). Three days later, media was removed, cells were rinsed with PBS, and fresh media without dox was added to the cells. For replication studies conducted for 7 days HepAD38 cells were plated in 6-well plates 1 day prior to scAAV-ZFN treatment without further passaging. As HepAD38 cell viability is significantly reduced upon prolonged culture, replication studies conducted for 14 days used HepAD38 cells that were plated in 60 mm dishes and passaged to new dishes with equalized cell numbers and fresh medium every 2 days to maintain cell viability.

### HBV droplet digital PCR (ddPCR) assay

Levels of HBV DNA in HepAD38-derived cellular DNA or supernatants were analyzed by ddPCR at the University of Washington, Department of Laboratory Medicine, Molecular Virology Laboratory using a laboratory-developed assay. DNA samples were extracted from cells or supernatant using the Roche MagNA Pure 96 system and total HBV DNA was quantified using primers specific for HBV ORF P/ORF S that were developed to amplify sequences from genotypes A–H. Deoxyinosine (bold) was incorporated in primers to allow for binding at heterogeneous sites and locked nucleic acids (underlined) were incorporated in the probe to stabilize structure and increase the melting temperature. HBV quantitation was done via a modification of the qPCR method described in [35], using the revised primers HBV-qPCR-F GATGT**I**TCTGCGGCGTT**I**TATC and HBV-qPCR-R GAGGACA**I**ACGGGCAACATAC, and probe HBV-qPCR-P 6FAM- CATCCTGCTGCTATGCCTC-BHQ1 in a duplex droplet digital PCR (ddPCR) assay for HBV and RPP30 on the QX100 ddPCR system (Bio-Rad Laboratories, Hercules, CA). RPP30 is a ribonuclease reference gene for cell count that exists at 2 copies/cell. The RPP30 primer and probe set were provided by Bio-Rad Laboratories and have the following sequences: RPP30for 5′-GATTTGGACCTGCGAGCG-3′, RPP30rev 5′-GCGGCTGTCTCCACAAGT-3′, RPP30probe 5′-Hex-TCTGACCTGAAGGCTCTGCGCG-BHQ1-3′

The ddPCR reaction mixture consisted of 12.5 µl of a 2X ddPCR Mastermix (Bio-Rad), 1.25 µl of each 20X primer-probe mix, and 10 µl of template DNA (previously digested with HindIII) in a final volume of 25 µl. Twenty µl of each reaction mixture was loaded onto a disposable plastic cartridge (Bio-Rad) with 70 ul of droplet generation oil (Bio-Rad) and placed in the droplet generator (Bio-Rad). The droplets generated from each sample were transferred to a 96-well PCR plate and PCR amplification was performed on a 2720 Thermal Cycler (Applied Biosystems, Carlsbad, CA) with the following conditions: 94°C for 10 minutes, 40 cycles of 94°C for 30 seconds and 60°C for 1 minute, followed by 98°C for 10 minutes and ending at 4°C. After amplification, the plate was loaded onto the droplet reader (Bio-Rad) and the droplets from each well of the plate were automatically read at a rate of 32 wells/hour. Data were analyzed with QuantaSoft analysis software and quantitation of target molecules presented as copies/µl of PCR reaction. For quantification of cellular HBV levels, values were divided by cellular RPP30 levels to calculate HBV copies/cell.

### Statistics

The Mann–Whitney test was used to compare HBV levels between HepAD38 treatment groups. A two-sided test of proportions was used to compare rates of targeting by the 3 ZFN pairs as determined by SMRT sequencing.

## Results

### HBV-specific ZFNs

In order to verify that HBV-specific ZFN pairs could have broad activity against multiple HBV genotypes, we analyzed sequence conservation and the relative evolutionary rates of each ZFN target site across 3847 HBV sequences found in Genbank (Figure 1B). ZFN target sites were highly conserved with only 1 (ZFN1, ZFN2) or 3 (ZFN3) differences found in the targeted DNA binding domains from the consensus sequence (Figure 1B, bold and underlined). A Rate4site analysis [36], which predicts the likelihood of evolution having occurred at a given position, showed that ZFN target sites 1 and 3 had the lowest levels of sequence evolution. Conservation of the target sites within sequences of genotypes A, B&C, or D was found to be high across the three genotype groups, except for ZFN2 with genotype A (Figure S1).

While designing HBV-ZFN pairs, sequences with similarity to the HBV target sites were searched for in the human genome to detect potential off-target sequences. No off-target sites were found with fewer than 7 mismatches from the HBV site for the three ZFN pairs chosen. The number of off-target sites with 7 or 8 mismatches was minimal (ZFN1: 0; ZFN2: 10; ZFN3: 2). ZFN2 was determined to have the most potential off-target sites. This was because the right zinc finger protein of ZFN2 contained only 5 zinc fingers, while all others contained 6 zinc fingers, resulting in a recognition sequence of 15 instead of 18 nt for the right half of the target site.

### HBV-ZFN activity in mammalian cells

**Figure 2:**
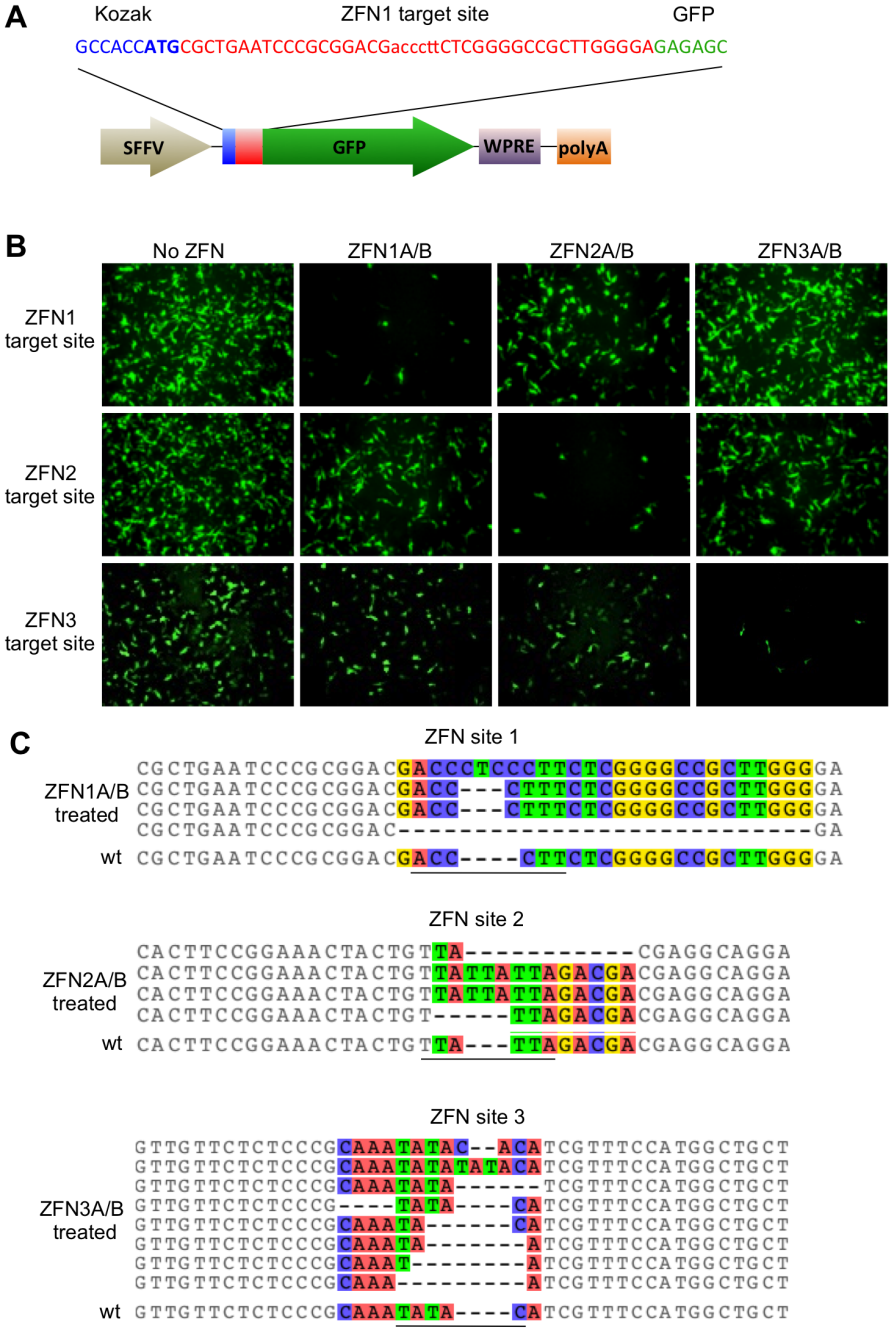
HBV-ZFN activity in mammalian cells. (***a***) HBV-ZFN target site/GFP reporter plasmid containing a ZFN target site (red) placed immediately after the GFP start codon (blue) and preceding the in-frame GFP ORF (green). The HBV-ZFN spacer is shown in lower case. (***b***) ZFN reporter constructs and expression plasmids for ZFN pairs 1–3 were transfected into HEK 293T cells. GFP expression was visualized at 72 hours post transfection. (***c***) ZFN-mediated target site disruptions within reporter constructs were amplified by PCR from the HEK 293T cells that had been transfected with ZFN-expressing plasmids as indicated. Indels found at each ZFN site are aligned with the wild type target sequence (wt) and nucleotides with differences in at least one sequence are shown in color. The spacer regions are underlined. GFP – green fluorescent protein; SFFV – spleen focus-forming virus promoter; WPRE – woodchuck hepatitis post-transcriptional regulatory element; polyA – polyadenylation signal; wt – wild type; ZFN – zinc finger nuclease.

To determine the activity of our HBV-ZFNs in mammalian cells we used a GFP reporter assay [16] to detect disruption of each ZFN target site. Three reporter plasmids each containing an HBV-ZFN target site between the GFP start codon and ORF (Figure 2A) were generated. The plasmids were individually transfected together with combinations of ZFN-expressing plasmids in order to monitor frame-shift mutagenesis of circular double-stranded DNA substrates in HEK 293T cells. A reduction in GFP expression was seen for each target site plasmid when treated with its corresponding ZFN pair (Figure 2B), suggesting that sequence-specific gene disruption was responsible. To confirm mutagenesis, the ZFN target sequences were amplified by PCR from cell DNA extracts. Clonal PCR amplicons were sequenced and both deletions and insertions were found within the target sites of all 3 ZFNs ranging from a 4 nt insertion to a 23 nt deletion (Figure 2C). Disruptive frame-shift mutations were introduced within the target sites of all reporter constructs specifically at the spacer regions where the ZFNs are expected to cleave. As such, we concluded that our HBV-ZFNs were able to efficiently disrupt gene expression in mammalian cells.

### scAAV-mediated delivery of HBV-ZFNs

**Figure 3:**
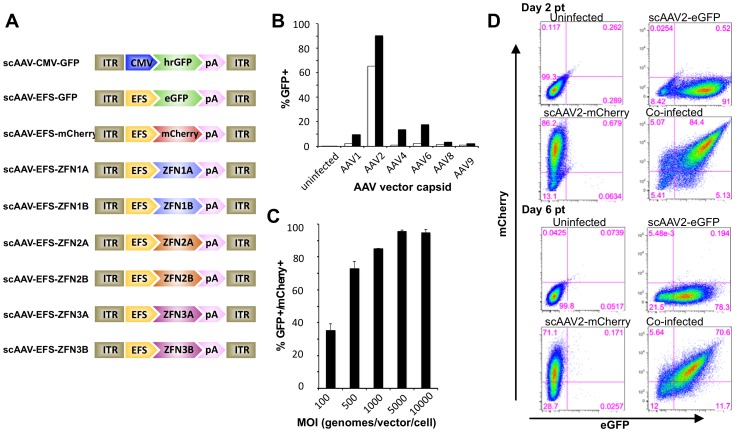
scAAV vector transduction of HepAD38 cells. (***a***) scAAV vector construct organization. (***b***) Cells were transduced with scAAV vectors containing a CMV-hrGFP-pA expression cassette at a MOI of 500 (open bars) or 5000 (closed bars) vector genomes/cell, or (***c***) were co-transduced with scAAV2-EFS-GFP and scAAV2-EFS-mCherry vectors at indicated MOI. Cells were analyzed for GFP and mCherry expression by flow cytometry at 72 hours post infection. (***d***) HepAD38 co-transduction persistence. Cells were transduced with scAAV2-EFS-eGFP and/or scAAV-EFS-mCherry at MOI 5000 genomes/vector/cell and co-transduction levels were monitored by flow cytometry at 2 and 6 days post transduction. ITR – inverted terminal repeat; CMV – cytomegalovirus immediate early promoter; EFS – elongation factor-1a short promoter; hrGFP – humanized Renilla green fluorescent protein; eGFP – enhanced green fluorescent protein; pA – polyadenylation signal; ZFN – zinc finger nuclease.

High levels of transgene delivery into target cells would be essential for therapeutic benefit. Viral vectors such as adeno-associated virus (AAV) vectors are capable of transducing hepatocytes both *in vitro* and *in vivo* at high levels without substantial toxicity. Self-complementary AAV (scAAV) vectors have been shown to achieve higher levels of transduction and greater transgene expression than single-stranded AAV (ssAAV) vectors [37]. Therefore, we generated a panel of reporter- or ZFN-expressing scAAV vector constructs to maximize delivery (Figure 3A). scAAV-mediated gene delivery was then analyzed in the HepAD38 cell line, an *in vitro* model for HBV replication, by flow cytometry. Depending on the application, different AAV capsids could be generated according to which serotype provides the most efficient delivery. Therefore, capsids from AAV serotypes 1, 2, 4, 6, 8 and 9 were all tested in order to determine which capsid would be most effective for transduction of the HepAD38 cell line. scAAV2 provided the most efficient gene delivery (Figure 3B).

To determine whether scAAV vectors could be used to deliver both halves of a ZFN to a significant number of hepatocytes, we co-infected HepAD38 cells with 2 scAAV reporter viruses (scAAV2-EFS-GFP and scAAV2-EFS-mCherry). Up to 96% of HepAD38 expressed both GFP and mCherry (Figure 3C). We observed significant co-expression even after 6 days post-transduction with over 70% of cells still positive for both GFP and mCherry (Figure 3D). These data suggest that scAAV can be used to efficiently deliver both halves of a therapeutic ZFN for a significant length of time.

### scAAV-HBV-ZFN-mediated cellular toxicity

**Figure 4:**
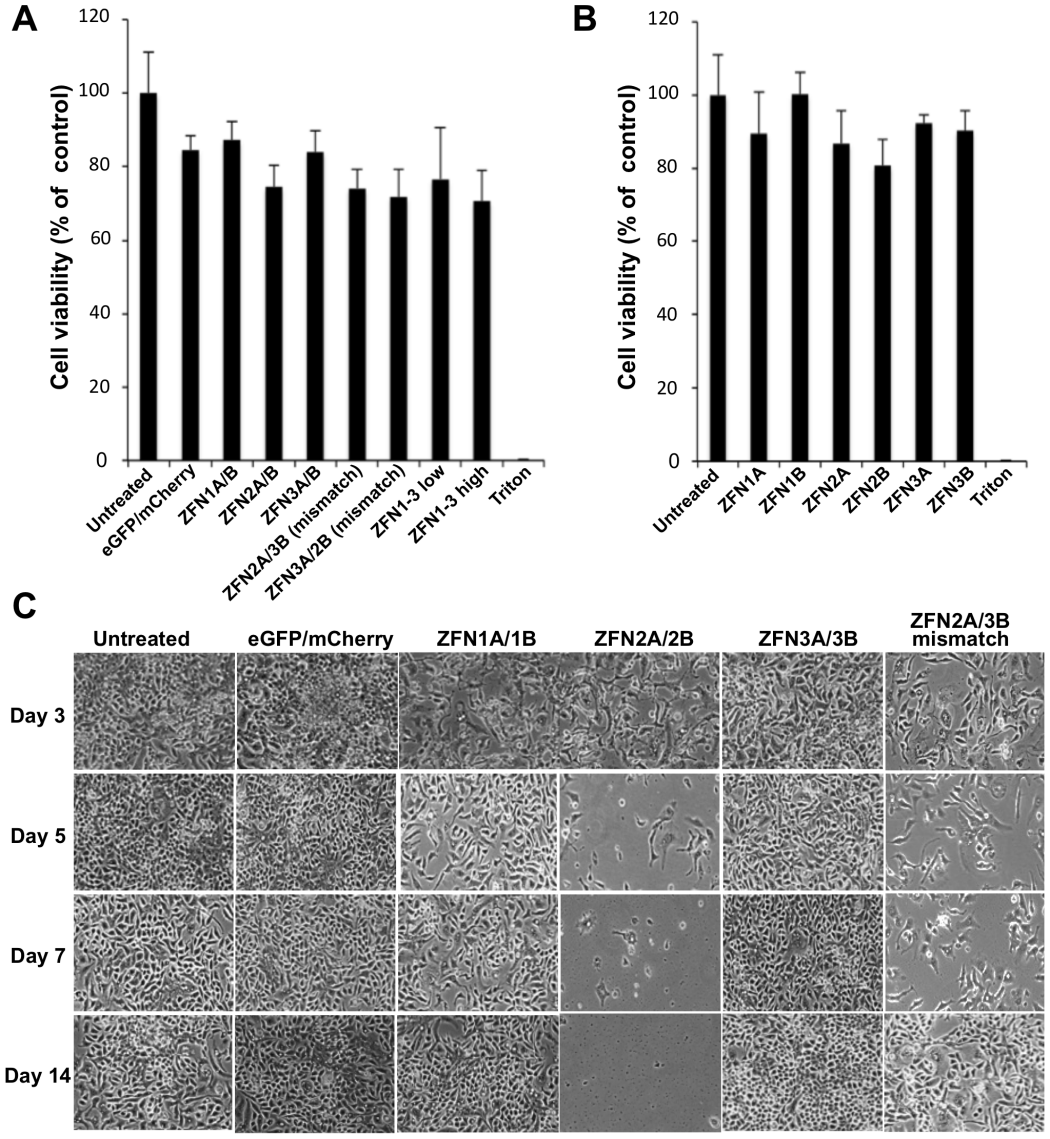
scAAV-ZFN induced cytotoxicity. (***a***) HepAD38 cells were transduced with scAAV2 vectors expressing GFP and mCherry reporter genes, ZFN pairs 1, 2 or 3, or mismatched ZFN pairs at a total MOI of 10000 genomes/cell, or all 3 ZFN pairs at a total MOI of 10000 (low) and 30000 (high) genomes/cell. At 48 hours post transduction, cell viability was measured by MTT assay and reported as percent of control. (***b***) HepAD38 cells transduced with scAAV2 vectors expressing individual ZFN half sites at a MOI of 5000 genomes/cell were also analyzed. (***c***) Untreated cells and cells treated with scAAV2 vectors expressing reporter genes, ZFN pairs 1, 2 or 3 or a mismatch ZFN pair were monitored for cell viability at 3, 5, 7 and 14 days post transduction. eGFP – enhanced green fluorescent protein; ZFN – zinc finger nuclease.

We analyzed the effects of transduction with scAAV-HBV-ZFN on HepAD38 cell viability. While scAAV infection at an MOI of 10000 genomes/cell reduced cell viability slightly, the viability of cells co-transduced with ZFN pairs containing ZFN2A and/or ZFN2B was below the other ZFN pairs (Figure 4A). Additionally, cells transduced with ZFN2A or ZFN2B had lower viability when compared to the other ZFNs individually from pair 1 or 3 (Figure 4B). In two-week-long viability assays, co-transduction with GFP and mCherry or ZFN pair 1 or 3 had no discernable effect on cell survival over 14 days in culture (Figure 4C). On the other hand, mismatch pair ZFN2A/3B caused reduced levels of cell survival, and ZFN pair 2 showed significant levels of toxicity at days 5 and 7 post-transduction (Figure 4C). By day 9 all cells in this treatment group had died. Thus, expression of either ZFN2A or ZFN2B alone reduced HepAD38 cell viability, and in combination their expression was lethal.

### scAAV-HBV-ZFN-mediated DNA mutations in HepAD38 cells

**Figure 5:**
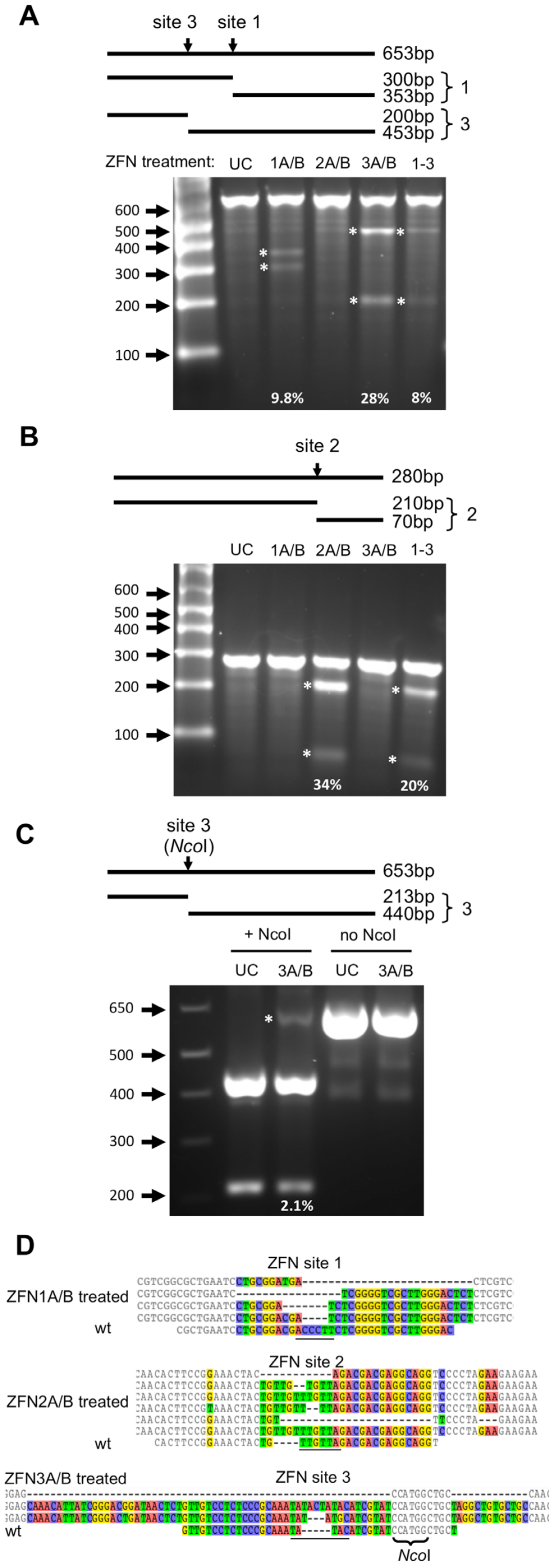
ZFN-induced target site disruption in HepAD38 cells. (***a, b***) Cells were transduced with scAAV2 vectors expressing ZFN pairs 1, 2, 3 or all three pairs together (1–3) at a total MOI of 10000 genomes/cell. The presence of mutations was analyzed in DNA isolated from transduced cells 72 hours later using the Surveyor nuclease assay. (***c***) For ZFN site 3, an analysis of DNA mutagenic events disrupting the internal NcoI cleavage site was also performed. Above the gel images, the sizes of PCR amplicons and the cleavage products produced upon Surveyor nuclease cleavage (indicating mutations at the indicated target site) or NcoI cleavage are shown. bp – base pairs; UC – untreated control; ZFN – zinc finger nuclease. Bands indicating mutations are highlighted with an asterisk and the percentage of ZFN-mediated DNA mutation for each targeted site is indicated. (***d***) DNA mutations that were detected at ZFN target sites 1, 2 and 3 within HepAD38 HBV sequences are shown above the wild-type ZFN site. Nucleotides with differences in at least one sequence are shown in color. Spacer regions are underlined. The rates at which DNA mutations were detected are listed in Table 1. wt – wild type.

Dox inhibits HBV replication in HepAD38 cells. While replication is stopped, the only substrate for HBV gene disruption is the single integrated copy of the HBV genome. Three days after ZFN delivery by scAAV2 vectors in cells incubated with dox, DNA extracted from the cells was analyzed for mutations through Surveyor nuclease digestion or NcoI digestion (Figure 5A–C). Surveyor analysis, a method for identifying DNA mutations at a specific site, showed that 9.8%, 34% and 28% of ZFN target sites 1, 2 and 3 were mutated, respectively, when treated with their corresponding ZFN pairs (Figure 5A–B, Table 1). ZFN site 3 contains an NcoI restriction enzyme site 10 nucleotides away from the center of the ZFN cleavage site, which could be lost upon large DNA deletions at the cleavage site (Figure 5D). After NcoI digestion of the ZFN3 target site PCR amplicon, quantification of undigested PCR product showed that 2.1% of the DNA was resistant to digestion by NcoI in ZFN3A/B-treated cells (Figure 5C). This indicated that the NcoI site was lost in a small fraction of HBV genomes following treatment with ZFN3A/B. HepAD38 cells were also treated with all 3 HBV-specific ZFNs at one-third the MOI for each ZFN pair, resulting in gene disruption in 20% and 8% of ZFN sites 2 and 3, respectively (Figure 5A–B). Clonal amplicon sequencing of the target sites revealed insertions and deletions at each of the ZFN sites (Figure 5D, Table 1) when treated with their corresponding ZFN pairs. Overall, a significant level of ZFN target site mutation was seen after HBV-ZFN treatment.

**Table 1:** Summary of HBV-ZFN-induced mutagenesis in HepAD38 cells.

ZFN site	ZFN treatment	Total mutations (colony sequencing)	Insertions (size nt)	Deletions (size nt)	Other	Surveyor assay (% indels)*
**1**	Uninfected	0/18				0%
	1A/B	5/20	0	4 (4, 7, 16, 26)	1 (HR)	9.8%
	2A/B					0%
	3A/B	0/21				
	2A/3B	0/17				
	all 1–3	0/62	0	0	0	0%
**2**	Uninfected	0/12				0%
	1A/B					0%
	2A/B	6/15	4 (2, 2, 4, 4)	2 (7, 23)	0	34%
	3A/B					0%
	2A/3B	0/20				
	all 1–3	5/64	4 (2, 3, 4, 4)	1 (29)	0	20%
**3**	Uninfected	0/18				0%
	1A/B	0/20				0%
	3A/B	3/21	2 (2, 5)	1 (69)	0	28%
	2A/3B	0/17				
	all 1–3	1/62	0	1 (16)	0	8%

*% indel  =  (1−(1−(a+b)/(a+b+c))∧0.5)*100; where a and b =  cut bands, c =  uncut band.

In order to analyze the ability of the ZFN pairs to target and mutate HBV cccDNA in HepAD38 cells, similar experiments were run in which prior to treatment with scAAV-HBV-ZFN, cccDNA production was induced by removing dox. Dox was then added at the same time as treatment with the scAAV-HBV-ZFN to prevent new cccDNA production and ensure that any mutations detected in cccDNA would not reflect newly synthesized cccDNA derived from mutated integrated HBV sequences. Cellular DNA extracts were treated with ATP-dependent Plasmid-safe DNase in order to isolate cccDNA. When Surveyor analysis or clonal PCR amplicon sequencing was performed, evidence of site-specific mutations could not be reproducibly detected (data not shown).

### Off-target mutagenesis

To analyze the fidelity of the ZFNs to their cognate HBV target sites, we selected seven potential off-target sites contained in the human genome with 9 or fewer mismatches from the HBV sites (Table S2). Using single molecule real time (SMRT) sequencing, we sequenced PCR amplicons from cell extracts following treatment with scAAV2-ZFNs. Reads were eliminated for being too low quality (LQ), the wrong size, possessing significant differences from the reference sequences, or for containing a single nucleotide indel in the target site spacer region. Amplicons for the genomic HBV on-target ZFN target sites were included in the sequencing run. These revealed frequencies of mutagenesis of 16%, 43%, and 24% for site 1, 2, and 3, respectively, when treated with their corresponding ZFN pair (Table 2, top). From the 9,290 filtered sequencing reads for the seven off-target sites treated with their corresponding ZFN pair (Table 2, bottom), the presence of indels larger than 1 nt were found in the target site of only 4 reads (Figure S2). Thus, we concluded that the HBV-specific ZFNs caused extremely minimal levels of off-target mutagenesis at these sites in the HepAD38 cell line.

**Table 2:** SMRT sequencing results showing on-target and off-target activity of HBV-ZFNs.

ZFN pair treatment	ampli-con	chromosome location	mis-match	reads	eliminated			remaining reads	total indels	inserts	deletions	other	targeting (%)
					LQ	poor align	single in-del in TS						
	**On-target HBV sites**												
Z1	HBV_Z1	HBV_1441	4	799	133	39	70	557	88	25	61	2	15.8*
Z2	HBV_Z2	HBV_2327	1	3274	177	38	288	2771	1200	594	433	173	43.3*
Z3	HBV_Z3	HBV_1334	2	314	71	12	8	223	53	21	30	2	23.8*
	**Off-target sites**												
Z1	Z1Ch14	chr14_74707199	9	2464	253	69	84	2058	1	0	1	0	0.05
	Z1Ch9	chr9_114246410	9	618	95	70	4	449	0	0	0	0	0
Z2	Z2Ch5	chr5_111607940	8	1729	196	51	24	1458	0	0	0	0	0
	Z2ChX	chrX_139173047	7	2084	194	52	56	1782	0	0	0	0	0
	Z2ChY, Z2Ch15	chrY_27576157, chr15_84923524	7	793	131	3	26	633	1	0	1	0	0.16
Z3	Z3Ch4	chr4_134502137	8	1279	118	20	6	1135	0	0	0	0	0
	Z3Ch15	chr15_34298524	8	2083	253	38	17	1775	2	1	1	0	0.11

LQ, low quality; TS, target site; *Significance between Z1 and Z3 (p<0.01), Z1 and Z2 (p<0.001), Z2 and Z3 (p<0.001).

### Inhibition of HBV replication after ZFN treatment of HepAD38 cells

**Figure 6:**
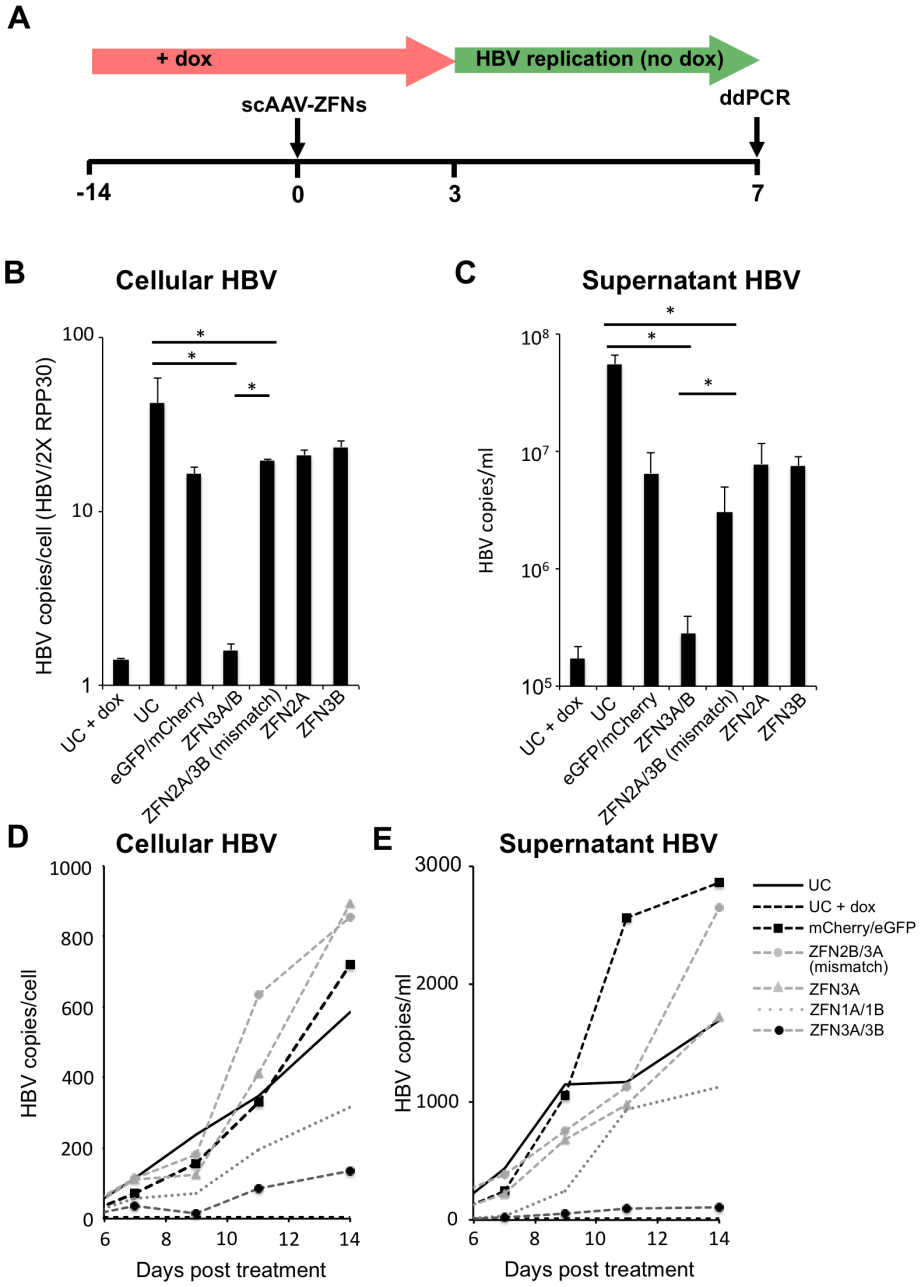
Levels of HBV DNA present in ZFN-treated HepAD38 cells and levels of secreted infectious HBV. (***a***) For experiments shown in panels ***b*** and ***c***, HepAD38 cells in the presence of dox were transduced with ZFN- or control-expressing scAAV2 vectors (total MOI 10000 genomes/cell) and 3 days later dox was removed from culture medium to enable HBV replication. Cells were left in culture for a further 7 days before HBV genomic levels were quantified in cells (***b***) and infectious HBV levels were quantified in supernatants (***c***) by ddPCR. (***d–e***) HepAD38 cells were passaged every two to three days over the course of 14 days following treatment with ZFN- or control-expressing scAAV vectors and monitored for cellular (***d***) and supernatant (***e***) HBV levels by ddPCR. ddPCR – droplet digital polymerase chain reaction; dox – doxycycline; eGFP – enhanced green fluorescent protein; scAAV – self-complementary adeno-associated virus; UC – untreated control; ZFN – zinc finger nuclease. *p≤0.05.

To test the effect of ZFNs on HBV replication, dox was removed 3 days after treatment with scAAV2-ZFNs (Figure 6A). Upon removal of dox, an intact HBV genome will initiate replication, producing high levels of cellular relaxed circular DNA (rcDNA) and secretion of new virions. Seven days later, we performed a sensitive droplet digital PCR assay (ddPCR) [38] to detect HBV levels in the cellular extracts and cell culture media (Figure 6B–C). Cellular HBV levels were normalized to the cell numbers as determined by the quantification of the housekeeping gene RPP30 to account for experimental variation. Removal of dox resulted in a 30-fold and 323-fold rise in HBV levels in cells and cell supernatants, respectively. We chose to test only ZFN pair 3 since it had produced high levels of targeted mutagenesis without affecting cell viability. When cells treated with ZFN pair 3 were analyzed following dox removal, we detected no significant increase in cellular or supernatant levels of HBV.

We next tested the duration of the antiviral effect of the ZFNs in HepAD38 during 14 days following dox removal and tested both ZFN pairs 1 and 3 (Figure 6D–E). Analysis of cellular and supernatant HBV levels showed that while treatment with ZFN pair 1 produced a mild reduction in HBV levels over 14 days, treatment with ZFN pair 3 produced a sustained suppression of HBV levels over the course of the experiment.

## Discussion

As a different approach from current anti-viral drug therapies, we are developing a strategy to eliminate replication-competent virus from chronic HBV patients. Here, we present data on the use of ZFNs to generate DSBs in unique DNA sequences within HBV genes. We predict that the introduction of targeted DSBs will result in mutagenesis within the HBV genes and prevent viral gene expression and viral replication. Previously, duck HBV and human HBV have been targeted with zinc finger proteins [39], [40] and ZFNs [17]. We have applied a similar technology toward targeting HBV and have utilized scAAV delivery vectors to achieve high levels of DNA mutagenesis with minimal off-target effects.

AAV vectors have safely been used to target liver cells in a number of animal models [41]–[43] and clinical trials [44], [45]. scAAV vectors achieve high transduction in multiple cell types and increased transgene expression over other AAV vectors such as single-stranded AAV [37], which have previously been used to effectively deliver ZFNs to the mouse liver [46]. However, the smaller packaging capacity of scAAV only allows for one ZFN subunit, thus requiring the co-transduction of two scAAV vectors to deliver a full ZFN pair. We were able to efficiently co-transduce over 95% of HepAd38 cells in culture, and scAAV co-transduction had a limited effect on cell viability at the experimental MOI. Additionally, AAV vectors of different serotypes can easily be constructed as needed depending on the application. *In vivo* transduction of close to 100% of mouse hepatocytes with AAV8 vectors has been shown at doses that produce no adverse toxicity [47]–[49]. Therefore, we anticipate that a high level of co-transduction can be achieved in the liver when the optimal vector is administered intravenously. The recent development of a chimeric AAV serotype with highly efficient human hepatocyte-tropic transduction when administered to a humanized murine model [50] will be very useful in future *in vivo* murine HBV experiments. Moreover, it has been reported that infection with HBV increases levels of AAV transduction in the liver both *in vitro* and *in vivo* [51].

In our ZFN target site/GFP reporter assay using transient plasmid transfections, ZFN pairs 1, 2 and 3 were able to efficiently knock down GFP expression in a manner that was dependent on site-specific gene disruption. However in HepAD38 cells, ZFN pairs 2 and 3 showed markedly higher targeted mutagenesis levels than ZFN pair 1. When all 3 ZFN pairs were delivered together this became even more apparent as no gene disruptions were detected at ZFN site 1 by Surveyor assay or clonal amplicon sequencing. This may be because ZFN pair 1 is a less efficient cleaver than ZFN pairs 2 and 3. Another possible explanation is that the ZFN site 1 in HepAD38 cells contains 4 mismatches from the target sequence for which the ZFN was designed whereas ZFN sites 2 and 3 only contain 1 and 2 mismatches, respectively (Figure 1B, blue letters). Alternatively, it could be because ZFN site 1 is less accessible than ZFN sites 2 and 3 due to epigenetic modification.

Previous studies have shown that the expression of ZFN pairs can lead to cellular toxicity that is likely mediated by non-specific cleavage at similar off-target binding sites [52], [53]. Although our ZFN pairs contain FokI nuclease domains that require heterodimerization for cleavage [26], which is thought to minimize the levels of ZFN-derived toxicity, toxicity still occurred for ZFN2. During our cell viability analysis we found that ZFN pair 2 was cytotoxic to HepAD38 cells, killing transduced cells by day 9 post-infection. Although ZFN2 did not exhibit off-target cleavage in the three sites chosen for analysis, this does not guarantee that there is not non-specific cleavage at other off-target sites in the human genome. ZFN targeting is known to involve context dependency for sequence binding [53], and off-target cleavage may occur more efficiently at sites with less sequence homology than the closest matches that are generated by tools such as PROGNOS. Particularly because of the smaller recognition sequence of ZFN2B compared to the other zinc finger recognition domains (15 vs. 18 nt), the quantity of near-matches throughout the genome is substantially higher for ZFN pair 2. An unbiased method for detecting off-target cleavage could potentially identify sites where cleavage occurs that we were unable to determine using PROGNOS, and potentially help to explain the toxic effect of ZFN2. The requirement for FokI to heterodimerize in order to cleave DNA makes it surprising that expression of ZFN2A or ZFN2B alone was toxic to cells. In fact, all groups containing one or both of ZFN2A or ZFN2B had reduced viability. Off-target cleavage by FokI homodimers could explain the toxicity. It is also possible that ZFN2A and ZFN2B are able to bind to specific DNA sequences and act as transcriptional inhibitors in a manner that induces cellular toxicity, without necessarily inducing DSBs.

To analyze the possibility of off-target mutagenic activity by the ZFN pairs, a high-throughput sequencing method was employed. SMRT sequencing allowed 10 separate PCR amplicons pooled on an individual chip to be sequenced resulting in over 10^4^ high quality reads. By aligning the sequence reads to reference sequences, indels at the HBV target sites or off-target sites could easily be detected. The degree of targeted mutagenesis observed in the three HBV sites was in agreement with earlier results obtained by clonal sequencing and the Surveyor assay (Tables 1 and 2). The higher number of reads provided by SMRT allowed more precise quantification of targeted mutagenesis, which showed significant differences between the three ZFN pairs, such that HBV mutagenesis by Z2 ≫ Z3 > Z1. These targeting efficiencies likely stem from the number of mismatches between the HepAD38 HBV sequence and the designed target sites (1, 2, and 4, for Z2, Z3 and Z1, respectively). All seven of the off-target sites showed either no or exceedingly small levels of mutagenesis. Based on the large number of reads, we can confidently say that the upper limit for off-target cleavage was 0.16% for any of the off-target sites. Moreover, the 4 reads indicating potential mutagenesis (out of 9290) all had small indels in regions with high sequence repetition (Figure S2) indicating that they could have been homopolymer-associated sequencing errors that happened to fall within the off-target site.

Despite only inducing mutations in 24–28% of genomic HBV target sites at 3 days post-delivery, ZFN pair 3 was able to knock down HBV replication and production of infectious HBV almost entirely by day 7. The Surveyor assay likely underestimates the true mutation rate [54], [55], so our actual rate of mutagenesis at day 3 may have been higher. It is also likely that in addition to disrupting genomic HBV sequences in HepAd38 cells, ZFNs can generate DSBs in both cccDNA and rcDNA species, which contributes to the knockdown of HBV replication seen in ZFN-pair-3-treated cells. However, it must be noted that we were unable to obtain reproducible evidence of cccDNA disruption in HepAD38 cells. Since we were able to achieve a complete and sustained knockdown of HBV replication with a single dose of scAAV2-ZFN3A/3B, we did not attempt to use multiple ZFN doses. In an *in vivo* model of HBV replication where a successful outcome may be more dependent on higher levels of HBV gene disruption, it may be desirable to give multiple doses of therapeutic AAV vector. We have used mathematical modeling to predict the likelihood of success for our approach in eliminating HBV infections and predicted that multiple therapeutic doses of HBV-specific enzymes will likely be needed [56]. Of note, at day 7 the mismatched pair of ZFN2A/3B showed a significant inhibition of HBV replication compared to the untreated control (Figure 6B–C), although not to the extent of ZFN pair 3. This knockdown was observed for all scAAV-treated samples, indicating that infection with the scAAV vector regardless of its payload has an inhibitory effect on HBV.

In a clinical setting, cccDNA would be the major target for HBV-specific ZFNs in hepatocytes of infected patients receiving concurrent antiviral drug therapy. It will be important to determine the efficiency with which site-specific endonucleases can target cccDNA. Despite reports that cccDNA can be targeted at certain sites by TALENs [22], [23], we believe the question of cccDNA cleavage and mutation efficiency requires continued attention. Bloom et al. published evidence of TALEN-mediated mutations in the cccDNA of HepG2.2.15 cells [23] and Chen et al. showed mutations in cccDNA in Huh7 cells transfected with linear HBV DNA [22]. However, neither group presented data showing cccDNA mutation in HepAD38 cells. It may be that the cccDNA present in HepAD38 cells is not amenable to cleavage, that it is not present in sufficient quantities to detect mutations, or that ZFNs are not as effective as TALENs. Our data would suggest that cccDNA is a more difficult substrate for targeted mutation than plasmid DNA or integrated DNA, and a more detailed analysis of its susceptibility to HBV-specific endonucleases would highly benefit this field of research. *In vivo* cccDNA takes on a highly organized chromatinized structure often referred to as the HBV minichromosome [57]–[59]. Due to the specific interactions between histones and cccDNA within the minichromosome it is unclear the degree to which target sites will be accessible for endonuclease-mediated cleavage. Aubert et al. [21] demonstrated that chromatin modifiers such as histone deacetylase inhibitors served to increase the mutagenic activity of targeted endonucleases on episomal herpes simplex virus DNA, and similar approaches might improve the mutagenesis of HBV cccDNA.

In order to detect HBV levels, we developed a sensitive ddPCR assay that can accurately detect sequences from HBV genotypes A–H. This involved using a primer/probe combination that is routinely used in a Taqman qPCR assay by the University of Washington Molecular Virology Laboratory to detect HBV in patient sera. This primer/probe combination is targeted to HBV ORF P/S and is used to detect infectious HBV in serum. In our HepAD38 cellular DNA extract samples, this assay will also detect genomic (integrated) HBV, rcDNA and cccDNA. Therefore, the experimental data must be interpreted with caution. In cellular DNA samples, we normalized HBV DNA values to the human gene RPP30, and thus values likely represent the total number of rcDNA and cccDNA molecules per cell.

In summary, we have developed an effective strategy to inhibit replication of HBV via HBV-specific ZFNs. We were able to efficiently deliver therapeutic ZFNs to HBV-infected hepatocytes using scAAV vectors, a platform for gene delivery that is adaptable to *in vivo*-scale HBV treatments, without substantial toxicity. Moreover, we have analyzed off-target activity of our three HBV-specific ZFNs at seven potential off-target sites without detecting any significant mutagenesis. The antiviral activity of our AAV-delivered therapeutic ZFNs was significant for ZFN pair 3, which reduced HBV DNA production to near-baseline levels and was sustained for up to two weeks. Taken together, these results support the feasibility of a ZFN-based strategy for targeting hepatitis B virus.

## Supporting Information

Figure S1 **HBV target site sequence heterogeneity.** Sequence heterogeneity for HBV-ZFN pairs 1–3 across 440 genotype A, 2233 genotype B&C, or 674 genotype D HBV sequences found in Genbank. For each ZFN pair the target sequence, logo plots, and consensus sequences obtained by aligning the target sequences with the genotype A, genotype B&C, or genotype D HBV sequences are shown. ZFN spacer nucleotides are highlighted in red and divergent nucleotides between the ZFN target site and the consensus sequence are bold and underlined. Single nucleotide polymorphisms present in the HepAD38 genomic HBV sequence are shown above each target site in blue. Logo plots and consensus sequences were obtained with the use of GeneiousPro. ZFN – zinc finger nuclease.
(TIF)

Figure S2 **Instances of indels in off-target site spacer regions.** Alignments with wt reference sequences of the 4 sequence reads of off-target sites containing indels in the spacer region. These were taken from a total of 9290 total reads of off-target sites obtained from PCR amplicons generated from DNA from cells that had been treated with ZFN-expressing scAAV2 vectors as indicated. Spacer regions are underlined. wt – wild type; ZFN – zinc finger nuclease.
(TIF)

Table S1 **PCR primer sets.**
(DOCX)

Table S2 **Three on-target and 7 off-target ZFN sites.** *Target sequence, red underlined nucleotides show spacer regions, lowercase letters show mismatches from HBV sites, blue nucleotides show variations contained in the HepAD38 HBV genome that are different from the designed ZFN sites.
(DOCX)
