# Supplementary figures and images for: AAV-Mediated Delivery of Zinc Finger Nucleases Targeting Hepatitis B Virus Inhibits Active Replication
Source: PLoS One. 2014 May 14;9(5):e97579. doi: 10.1371/journal.pone.0097579 (PMC4020843; doi:10.1371/journal.pone.0097579)

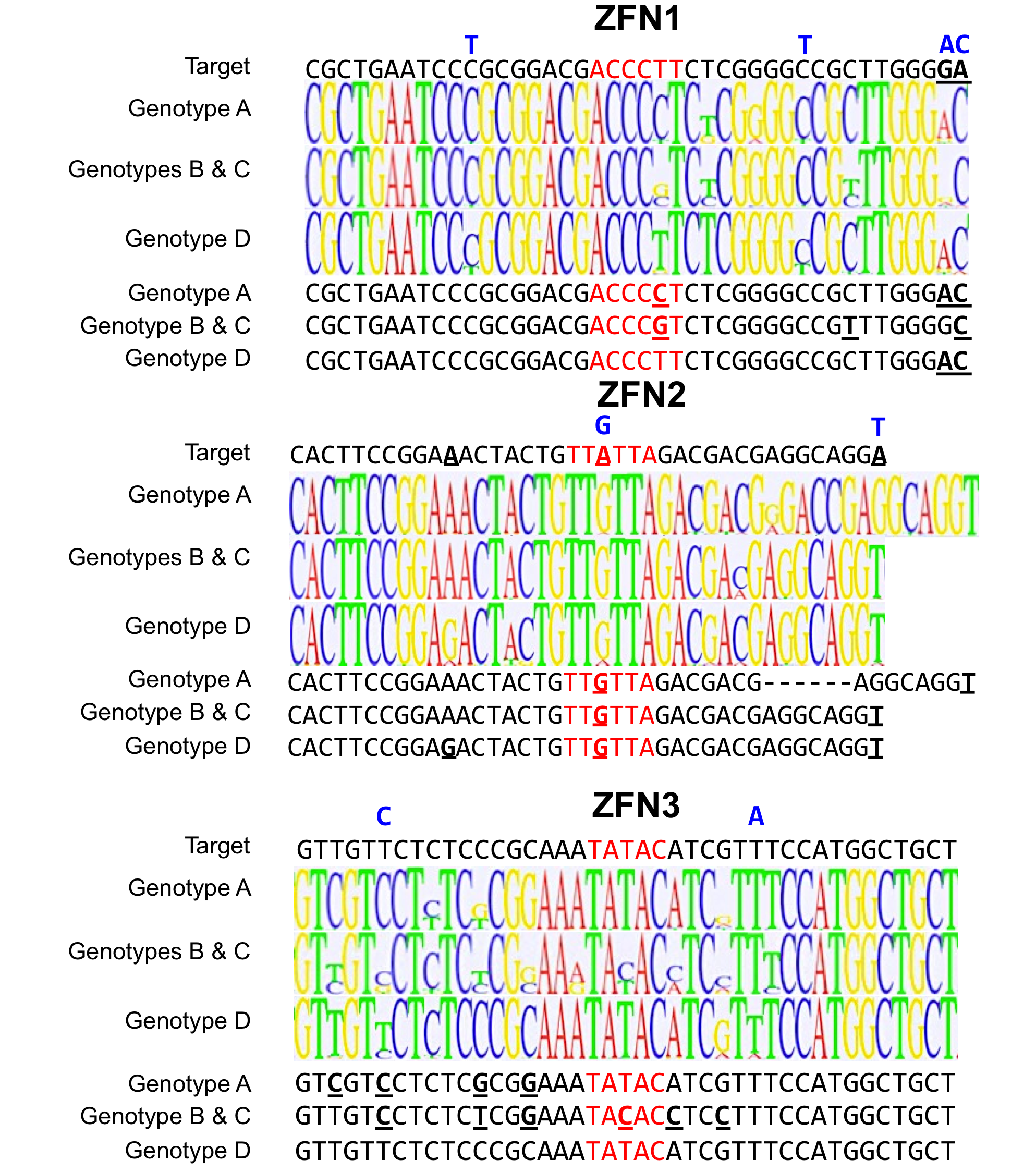

Supplement: Figure S1 — HBV target site sequence heterogeneity. Sequence heterogeneity for HBV-ZFN pairs 1–3 across 440 genotype A, 2233 genotype B&C, or 674 genotype D HBV sequences found in Genbank. For each ZFN pair the target sequence, logo plots, and consensus sequences obtained by aligning the target sequences with the genotype A, genotype B&C, or genotype D HBV sequences are shown. ZFN spacer nucleotides are highlighted in red and divergent nucleotides between the ZFN target site and the consensus sequence are bold and underlined. Single nucleotide polymorphisms present in the HepAD38 genomic HBV sequence are shown above each target site in blue. Logo plots and consensus sequences were obtained with the use of GeneiousPro. ZFN – zinc finger nuclease. (TIF) [file pone.0097579.s001.tif]

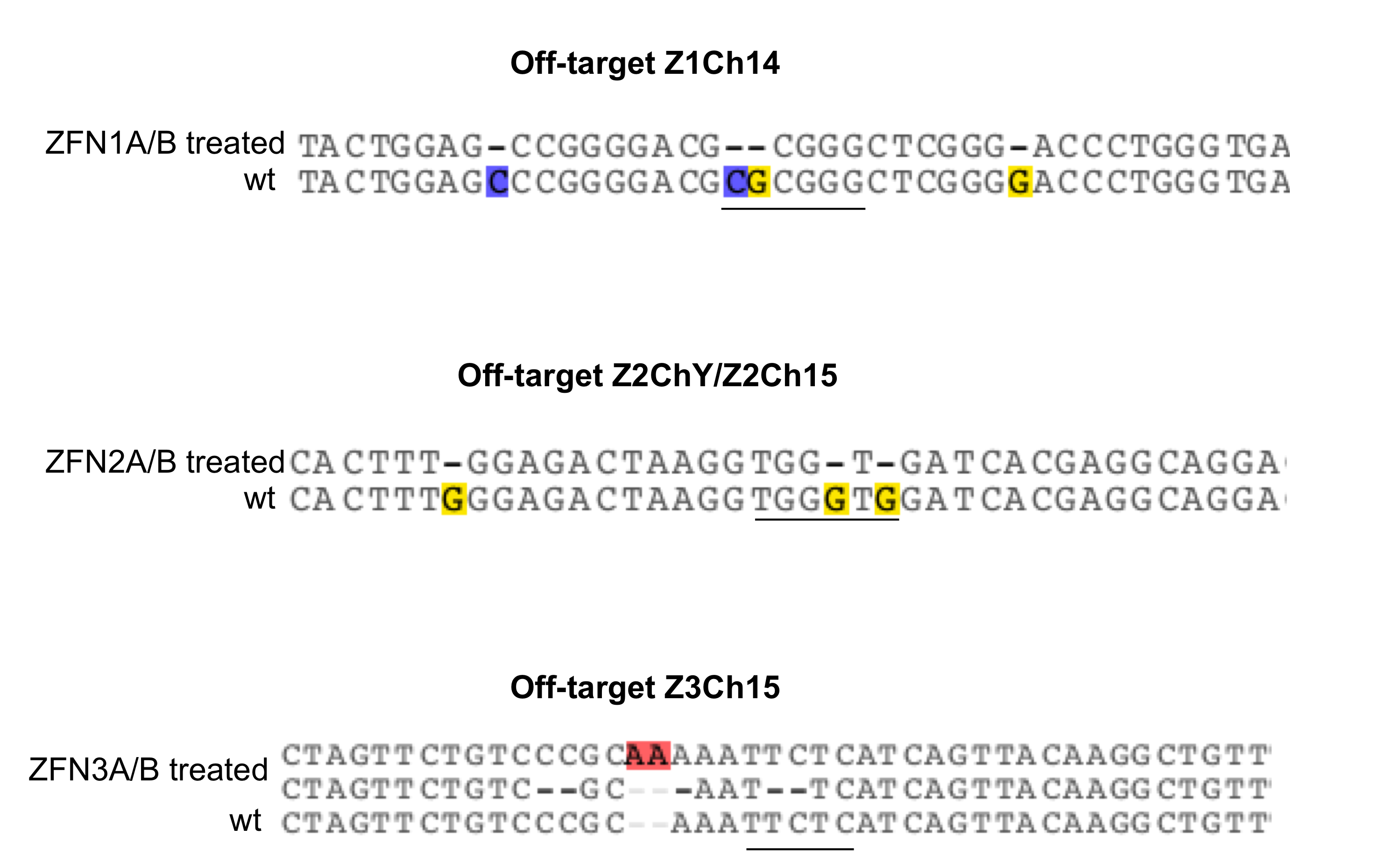

Supplement: Figure S2 — Instances of indels in off-target site spacer regions. Alignments with wt reference sequences of the 4 sequence reads of off-target sites containing indels in the spacer region. These were taken from a total of 9290 total reads of off-target sites obtained from PCR amplicons generated from DNA from cells that had been treated with ZFN-expressing scAAV2 vectors as indicated. Spacer regions are underlined. wt – wild type; ZFN – zinc finger nuclease. (TIF) [file pone.0097579.s002.tif]
