# Supplementary material for: AAV-Mediated Delivery of Zinc Finger Nucleases Targeting Hepatitis B Virus Inhibits Active Replication
Source: PLoS One. 2014 May 14;9(5):e97579. doi: 10.1371/journal.pone.0097579 (PMC4020843; doi:10.1371/journal.pone.0097579)
Supplement: Table S1 — PCR primer sets. (DOCX) [file pone.0097579.s003.docx]

**Table S1. PCR primer sets.**

| **PCR amplicon** | **Forward primer** | **Reverse primer** |
| --- | --- | --- |
| reporter plasmid TS (SFFV-F & TurboGFP-R) | GGAAGCTTGCCAAACAGGATATCTGCGGTGAGC | GACTAGTCGGGTAGGTGCCGAAGTGGTAGAAGC |
| HepAD38 ZFN1/3 (seq) | GGCTCCTCTGCCGATCCATA | ACACGGTCCGGCAGATGAGA |
| HepAD38 ZFN2 (seq) | AGTGTGGATTCGCACTCCTC | GCGGCGATTGAGACCTTCGT |
| Surveyor ZFN1/3 (1162-F & 1814-R) | AACGGCCAGGTCTGTGCCAA | GTGCTGGTGCGCAGACCAAT |
| Surveyor ZFN2  (2137-F & 2416-R) | CCAGCATCTAGAGACCTAGT | GCGGCGATTGAGACCTTCGT |
| pscAAV-EF1α  (EFS-F & -R) | GAGATCGGCGCGCCGGCTCCGGTGCCCGTCAGTGGGCAG | GAGATCCTCGAGACCTGTGTTCTGGCGGCAAACCCGT |
| pscAAV-mCherry  (mCherry-1 & -2) | GAGATCAGATCTCTCGAGGCCGCCACCATGGTGAGCAAGGGCGAGGAGGATAACATG | GAGATCGCGGCCGCTCACTTGTACAGCTCGTCCATGCCGCCGGT |
| pscAAV-ZFN-A  (ZFN-F & ZFN-R1) | GAGATCAAGCTTGCCGCCACCATGGACTACAAAGACCATGACGGTGATTA | GAGATCGCGGCCGCTCAAGATCTGAAGTTGATCTCGCCGTTGTTGAAC |
| pscAAV-ZFN-B  (ZFN-F & ZFN-R2) | GAGATCAAGCTTGCCGCCACCATGGACTACAAAGACCATGACGGTGATTA | GAGATCGCGGCCGCTCAGAAGTTGATCTCGCCGTTGTTGAACTTGCG |
| scAAV qPCR  (hrGFP-F & -R) | CCTTCGCCTTCGACATCCTG | CGCGGTACACGAACATCTCC |
| scAAV qPCR  (eGFP-F & -R) | ACGACGGCAACTACAAGAC | GGCGGATCTTGAAGTTCACC |
| scAAV qPCR (mCherry-F & mCherry-R) | CGCCAAGCTGAAGGTGACCA | AGATGAACTCGCCGTCCTGC |
| scAAV qPCR  (FokI-F & FokI-R) | GCGGCACAAGCTGAAGTACG | GATCACGCCGTAATCGATGG |
| SMRT Z1_HBV | GCGCTCTGTGTGCAGCAACGGCCAGGTCTGTGCCAA | GTGCTGGTGCGCAGACCAAT |
| SMRT Z2_HBV | CCAGCATCTAGAGACCTAGT | GCGGCGATTGAGACCTTCGT |
| SMRT Z3_HBV | CCTTGCCACAAGAACACA | TGGAGGCTTGAACAGTAGGA |
| SMRT Z1_Ch14 | GCGCTGGTCAACTGGGGTTT | GCACAATGCGTCCCTACTCG |
| SMRT Z1_Ch9 | CTCTTCCTTTGGACAGGCGT | GTCTGTGCGGCCGAATCC |
| SMRT Z2_Ch5 | GCACGAGAAGTTCCCTGTAGC | GCTGTAACCCGGTCTTTTTACTCC |
| SMRT Z2_ChX | CAGAATCCACTCTCCACCCC | AGGCAGTCTGGACCCTAGCA |
| SMRT Z2_ChY | AGAAACTGGTGAAAAGCAATCGT | GGTGGAGCTTGCCATGATCTG |
| SMRT Z3_Ch4 | CCTTCTCACCTTCCCAGTTTACC | TGTGGACCAAAATGCTCTCCT |
| SMRT Z3_Ch15 | AGAATGGCGTGAATCCAGGAGG | GTCTCCCACTTTCCATTTCAGTGC |
