# Supplementary material for: AAV-Mediated Delivery of Zinc Finger Nucleases Targeting Hepatitis B Virus Inhibits Active Replication
Source: PLoS One. 2014 May 14;9(5):e97579. doi: 10.1371/journal.pone.0097579 (PMC4020843; doi:10.1371/journal.pone.0097579)
Supplement: Table S2 — Three on-target and 7 off-target ZFN sites. *Target sequence, red underlined nucleotides show spacer regions, lowercase letters show mismatches from HBV sites, blue nucleotides show variations contained in the HepAD38 HBV genome that are different from the designed ZFN sites. (DOCX) [file pone.0097579.s004.docx]

**Table S2. Three on-target and 7 off-target ZFN sites.**

| **ZFN pair treatment** | **amplicon** | **chromosome location** | **mis-matches** | **sequence*** |
| --- | --- | --- | --- | --- |
| Z1 | HBV_Z1 | HBV_1441 | 4 | CGCTGAATCC**t**GCGGACGACCCTTCTCGGGG**t**CGCTTGGG**ac** |
|  | Z1Ch14 | chr14_74707199 | 9 | **ta**CTG**g**A**g**CCCG**g**GGACGCGCGGGCTCGGGG**a**C**c**CT**g**GG**t**GA |
|  | Z1Ch9 | chr9_114246410 | 9 | **gt**CT**cc**AT**g**CCGCGGACGCTGCGCTCGG**c**GCCGC**ga**GG**t**GA |
| Z2 | HBV_Z2 | HBV_2327 | 1 | CACTTCCGGAAACTACTGTT**g**TTAGACGACGAGGCAGG**t** |
|  | Z2Ch5 | chr5_111607940 | 8 | **t**ACT**c**CCGG**tt**ACT**c**CTGCTTGGGA**g**G**ct**GAGGCAGGA |
|  | Z2ChX | chrX_139173047 | 7 | CACTT**gg**GGAAACT**gag**GCCCAAGA**a**GACGAGGCAGG**c** |
|  | Z2ChY  Z2Ch15 | chrY_27576157  chr15_84923524 | 7 | CACTT**tg**GGA**g**ACTA**ag**GTGGGTGGA**tc**ACGAGGCAGGA |
| Z3 | HBV_Z3 | HBV_1334 | 2 | GTTGT**c**CTCTCCCGCAAATATACATCGT**a**TCCATGGCTGCT |
|  | Z3Ch4 | chr4_134502137 | 8 | **a**TTGTTCT**g**TCC**tg**AA**c**CCAGACAT**a**G**c**TTCCA**g**GGCTGCT |
|  | Z3Ch15 | chr15_34298524 | 8 | **c**T**a**GTTCT**g**TCCCGCAAATTCTCATC**ag**TT**a**CA**a**GGCTG**t**T |

*Target sequence, red underlined nucleotides show spacer regions, lowercase letters show mismatches from HBV sites, blue nucleotides show variations contained in the HepAD38 HBV genome that are different from the designed ZFN sites.
